# Evaluation of Indigenous Yeasts Screened from Chinese Vineyards as Potential Starters for Improving Wine Aroma

**DOI:** 10.3390/foods12163073

**Published:** 2023-08-16

**Authors:** Xiaoxin Ge, Jie Wang, Xiaodi Wang, Yaqiong Liu, Chao Dang, Ran Suo, Jianfeng Sun

**Affiliations:** 1 College of Food Science and Technology, Hebei Agricultural University, Baoding 071000, China; gxx2891@163.com (X.G.);; 2 Hebei Technology Innovation Center of Agricultural Products Processing, Baoding 071001, China

**Keywords:** *Saccharomyces cerevisiae*, non-*Saccharomyces*, wine, stress tolerance, enzymatic activity, volatile compounds

## Abstract

Exploitation of the biodiversity of native wine yeast is a means of modifying the sensory characteristics of wine. Samples from different regions in China were analysed to screen native isolates as potential starter cultures. Through morphological and molecular biological analyses, we found six species, belonging to four genera (*Hanseniaspora*, *Saccharomyces*, *Rhodotorula* and *Metschnikowia*). These species were subjected to stress tolerance assays (ethanol, glucose, SO_2_ and pH), enzymatic activity tests (sulphite reductase activity, β-glucosidase activity and protease activity) and fermentation tests. *Saccharomyces cerevisiae* showed a high tolerance to ethanol and completed fermentation independently. *Hanseniaspora* demonstrated good enzymatic activity and completed sequential fermentation. The fermentation experiment showed that the PCT4 strain had the best aroma complexity. This study provides a reference for selecting new starters from the perspective of flavour enzymes and tolerance and diversifying the sensory quality of wines from the region.

## 1. Introduction

The aroma characteristics of wine play a crucial role in determining the quality of wine and the wine-consuming experience. Wine is the product of many complex interactions among multiple microorganisms, which can produce different kinds of active flavour compounds during fermentation and play an undisputable role in the development of wine aroma [1,2]. Commercial *Saccharomyces* yeasts are preferred for wine production to ensure the stability of wine quality and standardise the process of fermentation [3,4]. However, the use of a limited number of commercial yeasts could weaken the distinction, regionality and uniqueness of wine from different regions [5,6]. Therefore, interest has recently been rising in indigenous yeast starters for producing wines with distinct and desirable characteristics [7,8,9].

Non-*Saccharomyces* yeasts originate mainly from the vineyard environment, including the soil and surface of grape [10]. However, *Saccharomyces* yeasts have less content on the surface of healthy fruits, and higher content in damaged fruits and natural fermenting must [11,12]. The number and variability of indigenous yeast species are highly influenced by several environmental factors, such as soil, grape variety and technological practices [13,14]. Different screening environments can be used to obtain yeast with special characteristics, making it possible to brew individualistic wine and influences the preferences for particular qualities [15]. China is currently one of the countries with the fastest growth in wine consumption in the world, and has more than 10 wine-producing regions [16]. However, studies on the selection and application of wine microorganisms are still very limited [16].

Non-*Saccharomyces* yeasts dominate the early stage of alcohol fermentation because they are present in higher numbers on grapes and winery equipment and are generally more cold tolerant than *Saccharomyces* yeast. They are replaced gradually by *Saccharomyces* spp. during alcoholic fermentation because *Saccharomyces* are more tolerant to hard conditions, especially ethanol [17]. However, recent discoveries in fermentation practice have been noted suggesting that some non-*Saccharomyces* yeasts can produce positive characteristics that are absent in *Saccharomyces* and may even improve the organoleptic profile of wine [18,19,20,21]. Therefore, sequential fermentation is usually used to ensure smooth fermentation and improve the quality of wine [22]. At the same time, to ensure the viability and positive influence of diverse yeasts in fermentation, it is necessary to pass appropriate screening to confirm whether a selected yeast can add desirable flavour attributes to the wine and serve as new starter [23].

The activity of related enzymes produced by yeasts has a significant effect on wine quality [24]. Glycoside precursors in wine are hydrolysed into terpene compounds under the action of β-glucosidase, thereby improving the aroma of the wine [25]. Proteases can promote the clarification of fruit juice to improve wine processing, and the products of protease degradation may be used as substrates for yeast growth [26]. Hydrogen sulphite has a negative organoleptic effect on wine due to the formation of off-flavours [27]. Therefore, yeast with beneficial properties can be screened from the perspective of flavour enzymes to improve the sensory characteristics of wine.

This work aimed to isolate desirable native yeasts from grapes, vineyard soil and different stages of fermentation in four representative grape-producing areas in China. Screenings were conducted using stress tolerance and enzyme activities (sulphite reductase, β-glucosidase and protease) to isolate yeasts for use as starter cultures. Single and sequential inoculation of selected indigenous yeasts was conducted to evaluate their contribution to the formation of volatile compounds and the fermentation kinetics of Cabernet Sauvignon wine.

## 2. Materials and Methods

### 2.1. Yeast Strains

All samples used in this study were collected from grapes, soil and un-inoculated grape must fermentations at different stages (pre, mid and end). The majority of the samples were obtained from the wine region in Huailai (Hebei Province), and the rest were collected from Penglai (Shandong Province), Yili (Xinjiang Province) and Qingtongxia (Ningxia Province). The samples were preserved under refrigerated conditions.

Samples (2 g) were placed in YPD liquid medium (1% yeast extract, 2% peptone and 2% glucose) and incubated in a shaker at 28 °C and 150 rad/min for 48 h. About 1 mL of the suspension was serially diluted to 10^−6^, and 0.1 mL of the diluted suspension was inoculated on YPD agar medium (1% yeast extract, 2% peptone, 2% glucose and 2% agar) for the isolation of yeasts. After incubation (28 °C for 48 h), different colonies were selected according to colour, appearance and size on the YPD agar medium and inoculated on WL medium (Qingdao Hope Bio-Technology Co., Ltd., Qingdao, China) for preliminary strain classification [28]. The WL medium was combined with 100 mg/L chloramphenicol after sterilisation to prevent bacterial contaminants [29]. A single colony was streaked on new WL medium and sub-cultured for more than three generations to purify the strain and reduce the risk of contaminants. For long-term conservation, the isolated yeasts were included in the collection and frozen in 40% *v/v* glycerol at −80 °C.

### 2.2. Molecular Characterisation of the Isolates

Genomic DNA was extracted from all yeasts isolated using a PlantZol Kit (Beijing TransGen Biotech Co., Ltd., Beijing, China). Fungus identification was carried out using ITS-PCR, with fungus-specific primers ITS1 (5′-TCCGTAGGTGAACCTGCGG-3′) and ITS4 (5′-TCCTCCGCTTATTGATATGC-3′). The fungal PCR system contained 15 µL of 2×EasyTaq^®^ PCR SuperMix, 1 μL of DNA template, 1.0 μL of 10 μM PCR forward primer, 1.0 μL of 10 μM PCR reverse primer and 12 μL of ddH_2_O. The PCR amplification cycling conditions were as follows: 94 °C for 5 min, followed by 35 cycles (94 °C, 30 s; 54 °C, 30 s; and 72 °C, 1 min) of fungal denaturation and a final 7 min extension at 72 °C.

Gel electrophoresis was performed using 2% agarose gel to evaluate the quality of DNA. Sanger sequencing of the PCR products was performed on an Illumina MiSeq PE300 sequencing platform (San Diego, CA, USA). For each group, yeast species were identified by sequence comparison with the Genbank^®^NIH genetic sequence database (NCBI; https://blast.ncbi.nlm.nih.gov/Blast.cgi (accessed on 5 June 2022)) [5,28].

### 2.3. Evaluation of Environmental Tolerance of Strains

Phenotypic screening was conducted to isolate desirable species identified under different stress conditions. The yeasts were inoculated in YPD liquid medium and incubated at 28 °C in a shaker to reach the exponential phase.

Stress resistances were evaluated on YPD liquid medium with different conditions of ethanol, SO_2_, glucose and pH, considering the criteria for selection of non-*Saccharomyces* yeasts described in a previous report [30]. The isolates were grown in media with different concentrations of ethanol (2%, 4%, 6%, 8% and 10%, where the appropriate volume of anhydrous ethanol was added to the medium after sterilisation), SO_2_ (50, 100, 150, 200 and 250 mg/L in the form of 30% *w/w* potassium metabisulphite solution (K_2_S_2_O_5_) was added at natural pH [28]), glucose (20%, 30%, 40% and 50%), and pH (3.1, 3.3 and 4.3). Roughly 3% yeasts in 10 mL of YPD liquid medium with corresponding pressure were added to 15 mL tubes with loose screw caps and Durham tubes.

The strains were incubated at 28 °C for 3 days with constant agitation (150 rpm) in an orbital shaker. The growth of the screened yeasts under stress agents was assessed according to gas production in the Durham tube using the following scale: 4 (full gas in the Durham tube), 3 (2/3 gas), 2 (1/3 gas), 1 (1/5 gas) and 0 (no gas). Basic YPD liquid medium without stress agents was used as control.

### 2.4. Screening of Enzymatic Activities

#### 2.4.1. Sulphite Reductase Activity

The capacity of the isolates to produce different levels of hydrogen sulphide (H_2_S) was evaluated with BIGGY agar medium [31]. Yeast was added onto the surface of BIGGY agar medium in a 96-well plate and incubated at 28 °C for 3 days. The ability to produce H_2_S was evaluated by colour using the following scale: 3, white (no H_2_S production); 2, light brown or yellow; and 1, dark brown. The YPD medium without yeast was used as a negative control.

#### 2.4.2. β-Glucosidase Activity

β-Glucosidase activity was determined using a medium containing 0.05% ammonium, 0.3% esculin, 0.2% NaCl, 0.05% MgSO_4_·7H_2_O, 0.01% KH_2_PO_4_ and 2% agar. Yeast incubated at 28 °C over night was added onto the surface of the 96-well plate and incubated at 28 °C for 1 day. The presence of β-glucosidase activity was determined based on the colour of the medium by using the following scale: 0, white (no β-glucosidase production); 1, dark brown. The YPD medium without yeast was used as a negative control.

#### 2.4.3. Protease Activity

Protease activity was determined using a medium containing 0.3% malt extract, 0.3% yeast extract, 0.5% peptone, 1% glucose, 0.5% NaCl and 2% agar, as described by Comitini et al. [32]. The same volume of 10% skimmed milk liquid was prepared. After sterilisation, the two liquids were mixed to prepare a sterile medium. The yeast cultures were spot plated and incubated at 28 °C for 3 days. The presence of a clear zone indicated the protease production of yeasts and evaluated as follows: 0, no clear zone (no protease production) and 1, clear zone (with protease production).

### 2.5. Sequential and Single Fermentation of Isolated Yeasts

#### 2.5.1. Alcoholic Fermentation

Sequential fermentation and single-culture fermentation were conducted to assess the fermentation ability of the isolated yeasts. The juice (pH 3.3, total sugar: 265 g/L glucose; total acidity: 8.9 g/L tartaric acids) was made from Cabernet Sauvignon grapes harvested in 2021 from the Hiailai region. Flasks were inoculated with 10^6^ cells/mL of each isolated yeast. Sequential fermentation was performed by inoculating non*Saccharomyces* yeasts into 250 mL of pasteurised musts at 25 °C for 48 h, followed by the inoculation of commercial *S. cerevisiae* CEC01 (0.2 g/L, Angel Yeast Co., Shanghai, China). Commercial non-*Saccharomyces* yeast NSD (*Torulaspora* species, 0.2 g/L, Angel Yeast Co., Shanghai, China), as a control, was used to compare with the isolated non-*Saccharomyces* yeasts in terms of fermentation rate and characteristics. Single-culture fermentation was carried out by inoculating isolated *S. cerevisiae* for fermentation and CEC01 as control. The growth rate of strains was monitored by weight loss as an estimate of CO_2_ production every 24 h until the end of fermentation. The lack of weight change in the wine samples indicated the end of fermentation.

#### 2.5.2. Volatile Compounds by HS-SPME-GC–MS

Volatile compounds in wine samples were determined by headspace–solid-phase micro-extraction–gas chromatography with mass spectrometry (HS-SPME-GC–MS). Before analysis, 10 μL of 3-octanol (300 mg/L) as an internal standard and 2 g of NaCl were added to a headspace bottle containing 8 mL of the wine sample. The bottle was vortexed for more than 3 s, incubated in a water bath at 40 °C for 15 min and extracted by SPME fibre headspace for 40 min. The analytes extracted from the fibre were inserted through the GC injection port for analysis for 8 min.

A glass capillary column was used: HP-Innowax (60 m × 0.25 mm × 0.25 μm). The carrier gas was helium, and the flow rate was 1 mL/min. The inlet temperature was 240 °C, and the split–splitless mode was adopted. The temperature program of the column oven was as follows: 3 °C/min to 80 °C; 80 °C for 6 min; and 5 °C/min to 240 °C. The ion source temperature was 230 °C, the impact energy was 70 eV, and the mass spectral scanning range was 40–350 m/z. The resulting mass spectrum was compared with the NIST 14 spectral library, and components that matched more than 80% of the results were analysed. Volatile compounds were quantified using an internal standard method.

#### 2.5.3. Oenological Parameters

Total acidity, total reducing sugars, pH, and the ethanol content of the fermentation liquid were determined according to the National Standard of the People’s Republic of China (GB/T15038-2006) [33]. The pH was measured with a digital pH meter, total acid was calculated by tartaric acid, ethanol was determined by distillation and reducing sugar was evaluated by titration with Fehling reagent [34].

## 3. Results and Discussion

### 3.1. Isolation and Characterisation of Yeasts

The climates and environments of different regions vary, so the types and quantities of yeast suitable for growth may not be the same [35,36]. A total of 79 samples were collected from eight different locations distributed among the four representative regions of China. The samples were used to isolate yeasts with excellent fermentation characteristics. A collection of 31 yeasts were isolated belonging to *Hanseniaspora*, *Saccharomyces*, *Rhodotorula* and *Metschnikowia* (Table 1). Most of the yeast samples were obtained from spontaneous fermentation, and fewer species were collected from the grape surface and soil. The screening results showed that the number of non-*Saccharomyces* species was higher than that of *S. cerevisiae*, consistent with the study of Borren [18].

*Hanseniaspora* species comprised the most abundant yeasts (55% of the yeasts) and were found in vineyards and grapes, consistent with the report of Portillo, M.d.C [37]. *Hanseniaspora* has low fermentation ability, but plays an important role in the production of volatile compounds in wine [38]. For example, *Hanseniaspora vineae* can increase the content of 2-phenylethyl acetate during fermentation, providing wine with floral and fruity aromas [39]. The second most abundant yeast was *Saccharomyces*, representing 19% of the yeasts; it was mostly found in the middle and latter stages of spontaneous fermentation and at very low abundance on berries (Table 1). *Saccharomyces* had good tolerance to different fermentation factors, especially to ethanol; as such, it could complete fermentation alone or be combined with non-*Saccharomyces* yeasts to complete sequential fermentation. Yeasts present in lower numbers included *Metschnikowia* (16%) and *Rhodotorula* (10%). The screened *Metschikowia* yeast mainly existed in fermented grape juice and represented 29% of grape juice; similar results have been reported by previous studies [14,40]. Chemical and sensory analyses showed that wine produced by *Rhodotorula mucillaginosa* WLR12 had more floral fragrance and some sweet and ripe fruit flavours compared with wine produced using commercial yeast [41].

Based on morphological observation and molecular biology identification, the isolate with flat, dark green colonies and apiculate cells on WL medium could be *Hanseniaspora* and the typical cream-coloured, round and unboned colonies could be related to *Saccharomyces* [31]. The isolate that was white or cream coloured, round on the surface, and with red-brownish pigment on the bottom could likely be associated with *Metschnikowia* yeast. Colonies of *Rhodotorula* might appear red, circular, and smooth in shape.

### 3.2. Features of Indigenous Yeasts

#### 3.2.1. Environmental Stress Tolerance Analysis

**Figure 1:**
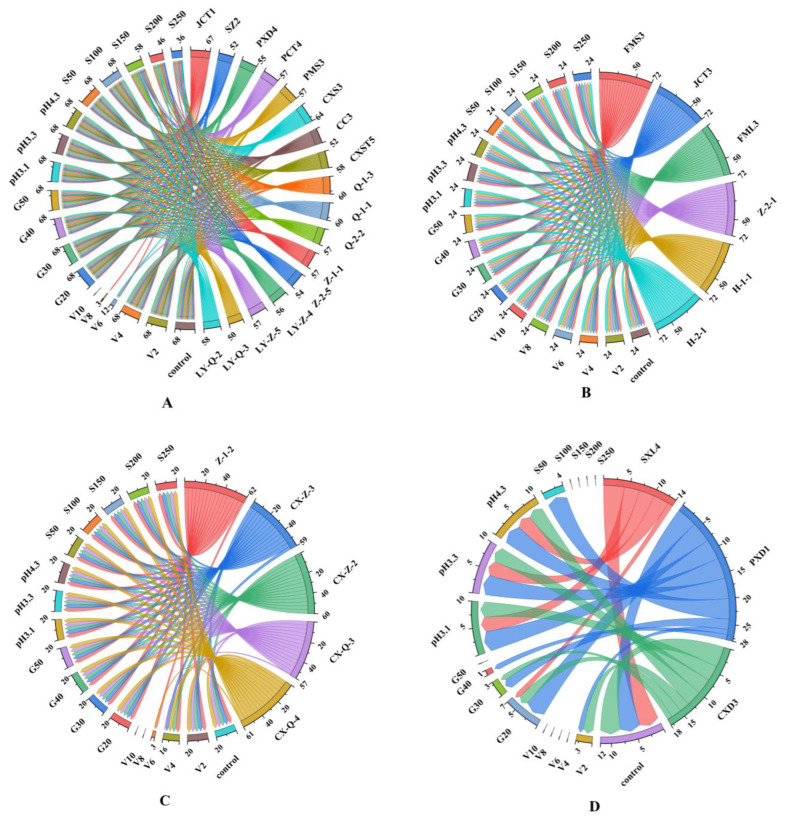
Chordal diagram: the tolerance of different genera of yeast under selective pressures. (**A**) *Hanseniaspora* genus; (**B**) *Saccharomyces* genus; (**C**) *Metschnikowia* genus; (**D**) *Rhodotorula* genus. V2–V10: 2–10% concentrations of ethanol; G20–G50: 20–50% concentrations of glucose; S50–S250: 50–250 mg/L concentrations of free SO_2_. The different colours in the figure correspond to different yeasts and pressure conditions. The left side of the chordal diagram presents the scores of different yeasts under different pressure conditions, and the right side presents the total score of tolerance of different strains under different pressure conditions. Detailed data can be found in Table S1.

The growth of yeast is affected by conditions such as pH, temperature, ethanol concentration, SO_2_ concentration and osmotic pressure during fermentation. Therefore, the tolerance of screened yeast strains to environmental stresses should be evaluated for their future use as starters in wine production. Strains isolated from different regions included 17 isolates of *Hanseniaspora*, 6 isolates of *Saccharomyces*, 5 isolates of *Metschnikowia* and 3 isolates of *Rhodotorula*, which were used for further analysis. Figure 1 and Table S1 show the performance of different yeasts under different pressure conditions.

According to the scoring standard, the total score of *S. cerevisiae* under all pressure conditions was 76, which was significantly higher than that of non-*Saccharomyces* yeasts, indicating the good tolerance of *S. cerevisiae*. Under all pressure conditions, the scores of *Hanseniaspora*, *Metschnikowia* and *Rhodotorula* were 54–71, 61–68 and 15–20, respectively, indicating that *Rhodotorula* had the worst tolerance. In addition, the ethanol tolerance of *S. cerevisiae* was better than that of non-*Saccharomyces* yeast. Further testing found that six strains of *S. cerevisiae* had ethanol tolerance of 12%, four strains had ethanol tolerance of 14% and one strain had ethanol tolerance of 16% (Table S1), which is consistent with the report of Liu [6]. Figure 1A shows the total scores of *Hanseniaspora* yeast under V2–V10 (ethanol concentration of 2–10%), where the pressure conditions of 68, 68, 12, 3 and 0 indicate the gradual decrease in its ethanol tolerance. These results are similar to those of previous studies, where it was reported that most species of *Hanseniaspora* and *Metschnikowia* can tolerate ethanol concentrations up to 4–6% [32]. For the isolated *Rhodotorula* yeast (Figure 1D), slight growth was observed in all ethanol concentrations. These results indicate the existence of inter-genus differences among different genera of yeast.

The same genus of non-*Saccharomyces* yeasts had different responses under the same stress conditions. For example, *Hanseniaspora* yeast JCT1 survived in the presence of 8% ethanol, whereas other *Hanseniaspora* yeasts tolerated 4% ethanol only. However, the general variation trends of the responses of non-*Saccharomyces* yeasts to ethanol, glucose and SO_2_ tolerance were similar, that is, the tolerance decreased with increasing concentrations of these environmental factors (Table S1). For instance, with increasing SO_2_ concentration (150–250 mg/L), the growth ability of *Hanseniaspora* yeast (SZ2) decreased gradually. *Rhodotorula* yeast (PXD1) showed a slower proliferation rate with increasing glucose level. Most of the yeasts tolerated the stress conditions of glucose concentrations of 20–50%, pH 3.1–4.3 and SO_2_ levels of 50–150 mg/L.

The slow growth of certain yeasts could be due to the following factors. Yeast cell membranes are considered the primary target of ethanol stress; ethanol alters the organisation and permeability of cell membranes and functional proteins, thereby affecting glucose uptake and fermentation rates under winemaking conditions [42,43]. In the early stages of vigorous fermentation, an environment with acidic pH prevents the growth of certain yeast [44]. In addition, a previous work has shown that SO_2_ clearly inhibits the growth of non-*Saccharomyces* species during fermentation [45].

#### 3.2.2. Enzymatic Activity Analysis

**Figure 2:**
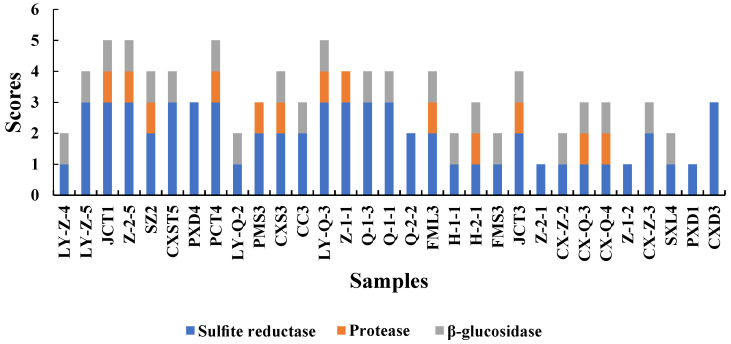
Sulphite reductase, protease and β-Glucosidase activities produced by isolated yeasts. These three enzymes were evalutated based on the scoring criteria presented in Section 2.4.

The aroma produced by yeast during fermentation is related to enzymes [18]. To improve the quality of wine, we screened yeasts with high-yield β-glucosidase and protease and low-yield H_2_S for winemaking. All enzymatic activity scores are shown in Figure 2.

Sulphite reductase is an important factor that should be considered in winemaking because it has a negative effect on wine quality [5]. Sulphite reductase was tested in BIGGY medium to evaluate the H_2_S potential of the screened yeasts [27]. Darker colour of the BIGGY medium indicated higher amounts of H_2_S produced, resulting in adverse effects on wine. All the screened *S. cerevisiae* samples produced sulphite reductase, but some of the strains produced low levels only. Among *Hanseniaspora* and *Rhodotorula,* a variability was observed, that is, they had no to low production of sulphite reductase, leading to higher quality of wine. Only CX-Z-3 produced a low level of H_2_S among *Metschnikowia* yeasts, similar to the findings of Binati [31].

Protease can promote the decomposition degradation of proteins into amino acids that are the precursors of aroma components. Moreover, the protease secreted by yeast can catalyse protein hydrolysis to release assimilated nitrogen source peptides, which may have beneficial effects on microorganisms during fermentation and prevent protein haze to maintain the stability of wine [5,46]. Among isolated *S. cerevisiae*, most yeasts (except for FMS3 and H-1-1) produced protease. Previous reports on *Metschnikowia* have indicated that it had proteases [31,38], while other studies have reported the opposite results [22]. In this study, proteolytic activity was detected in several isolates belonging to nearly half of the number of *Metschnikowia*. The mechanism of protease production remains unclear and should be further studied. Moreover, half of *Hanseniaspora* and yeasts were found to have protease production activity. Nevertheless, *Rhodotorula* lacked the ability to produce proteases.

One of the reasons that wine exhibited complex aromas is the presence of free and bound aroma precursors, which undergo enzymatic hydrolysis of β-glucosidases to obtain compounds such as glycosides and monoterpenes, which contributed to strong floral and fruity aroma [47]. The colour of the medium containing yeast was darker than that of the medium containing only YPD liquid medium, indicating that the yeast produced β-glucosidase. In this study, most of the species of *Hanseniaspora* (76%) and *Metschnikowia* (80%) had a positive result for β-glucosidase activity. Several studies have reported findings that β-glucosidase activity was higher in indigenous non-*Saccharomyces* yeasts than in *S. cerevisiae* [23,48], which is consistent with the present results. *S. cerevisiae* (except Z-2-1) had positive results for β-glucosidase, whereas only one *Rhodotorula* isolate (SXL4) exhibited extracellular β-glucosidase activity.

### 3.3. Fermentation Trials

#### 3.3.1. Fermentation Rate and Oenological Analysis

**Figure 3:**
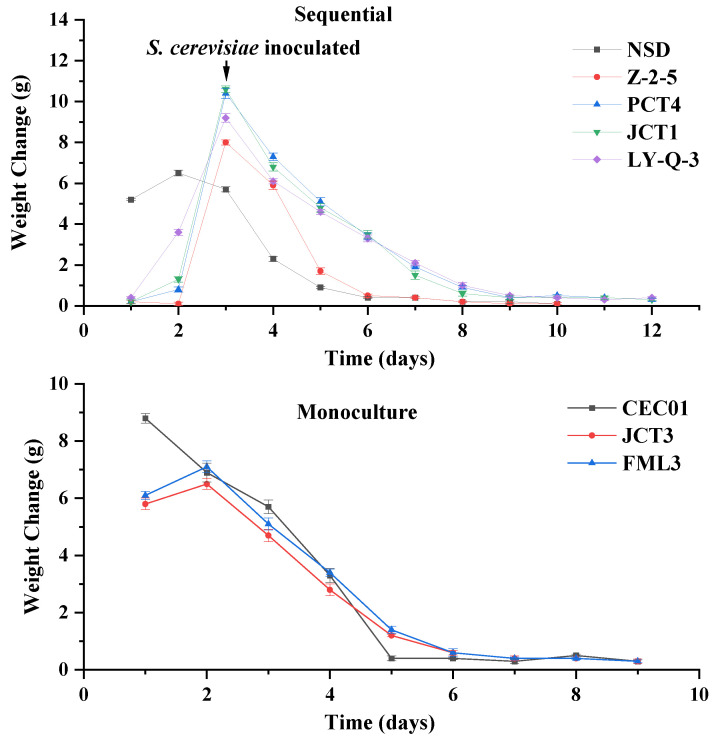
Fermentation rate of sequential and monoculture fermentation. Weight change is defined as the difference of weight between two adjacent days.

The isolates of *Hanseniaspora* (Z-2-5, PCT4, JCT1 and LY Q-3) and *S. cerevisiae* (FML3 and JCT3) showed better enzymatic activities and ethanol tolerance separately, and were further evaluated with respect to their influence on the wine aroma profile. Figure 3 shows the fermentation rate of native yeasts. The reducing sugar, ethanol, total acid and pH of the wine samples are shown in Table 2.

In monoculture fermentation, *S. cerevisiae* JCT3, FML3 and CEC01 completed fermentation independently (Figure 3). Compared with CEC01, the fermentation rate of the screened *S. cerevisiae* was slower in the early stage, but then approached that of CEC01 and fermentation was completed at the ninth day. Moreover, the ethanol content of isolated *S. cerevisiae* (FML3 and JCT3) was lower than that of commercial culture CEC01 (Table 2), indicating that FML3 and JCT3 could be used to produce wine with a low alcohol content. Moreover, their residual sugar content was higher than that of commercial *S. cerevisiae* CEC01, and their total acid content was significantly lower, leading to a reduction in the acidity of the wine.

In sequential fermentation using non-*Saccharomyces* yeasts and CEC01, it can be seen from Figure 3 that sequential fermentation took at least 10 days. This result indicates that the fermentation ability of sequential fermentation was worse than that of monoculture fermentation, which is consistent with the research of Ciani [49]. Following sequential inoculation with CEC01, Z-2-5 completed the fermentation the fastest (10 days) in isolated non-*Saccharomyces* yeast and equal to the cycle of NSD. In addition, Table 2 showed significant differences, especially for ethanol and pH, in wine samples inoculated with different yeasts. The concentrations of ethanol were more than 10% (*v*/*v*), except for Z-2-5. All of the screened non-*Saccharomyces* yeast, except for one culture (Z-2-5), had greater ability to produce ethanol than NSD in sequential fermentation. The pH of all samples was between 3 and 4, and Z-2-5 wine had the lowest pH, but the total acid content of Z-2-5 wine (8.47 g/L) was more than twice that of other samples. During fermentation, Z-2-5 yeast might produce more organic acids, decreasing the pH of wine, compared with the other yeast samples. The content of reducing sugar in wine samples ranged from 2.60 to 3.33, which could be due to the different nutrients utilised by different strains [50].

#### 3.3.2. Volatile Compound Analysis

A total of 55 volatile compounds, including alcohols, acids, esters and other types of compound, were identified at the end of fermentation with different yeasts. When comparing the content of volatile compounds in wines fermented by isolated yeasts, most of the compounds showed significant differences (*p* < 0.05). All data were obtained from pasteurized grape juice and could indicate the characteristics of the strains.

Esters are the most abundant compounds in almost all wine samples, and provide wine with fruity aromas [51,52]. The esters formed in the fermentation process are mainly acetic ester and fatty acid ethyl ester. Acetate esters are more dependent on the hydrolysis of amino acids or sugars with alcohol acetyltransferases (Atf1p and Atf2p) than as a precursor to higher alcohols [53]. Six acetates (isoamyl acetate, heptyl acetate, hexyl acetate, ethyl acetate, isobutyl acetate and decyl acetate) were detected in the wine samples. Heptyl acetate (44.50 μg/L) and isobutyl acetate (71.73 μg/L) were only detected in PCT4 and Z-2-5 groups, whereas ethyl acetate was found in Z-2-5 (7997.75 μg/L) and LY Q-3 (1552.18 μg/L). Compared with the two control groups, the wines of isolated yeasts had a higher number of decyl acetates, which produce floral and honey aromas [5,54]. Notably, the isoamyl acetate concentration produced by PCT4 was 16 times higher than that in the control group of NSD. In addition, yeasts produced fatty acid coenzyme A, which reacts with ethanol to produce ethyl esters of fatty acids [1]. In monoculture fermentation, ethyl dodecanoate and ethyl hexanoate were only found in isolated *S. cerevisiae*. In sequential fermentation, native non-*Saccharomyces* yeasts produced higher degrees of isoamyl acetate, ethyl octanoate, ethyl decanoate, ethyl 9-decenoate and decyl acetate compared with NSD. Native non-*Saccharomyces* yeast could produce ethyl dodecanoate (sweet and floral aroma), which was absent in NSD [54]. Compared with the other yeasts, PCT4 produced higher amounts of esters, especially ethyl 9-decenoate (rose aroma) and ethyl octanoate (pear aroma) [55].

Alcohols are the second most volatile compounds formed during fermentation, and they are mainly derived from yeast metabolism and hydrolysis of glycosides and esters. When the alcohol concentration is less than 400 mg/L, the wine exhibits a typical aroma and complex flavour [56]. Meanwhile, 17 alcohols were detected in the fermentation samples, and their contents were all lower than the standard. 2-Dodecanol (35.24 μg/L), 1-heptanol (102.69 μg/L) and 3-heptanol-5-methyl (329.96 μg/L) were specific to JCT3, PCT4 and NSD, respectively. The concentration of isobutyl alcohol was significantly higher in all non-*Saccharomyces* yeast samples compared with that in *S. cerevisiae*. Phenylethyl alcohol (rose aroma) was the most abundant among alcohols, and the content of NSD was more than 8 times that of Z-2-5 [54]. In sequential fermentation, FML3 and JCT3 contained isoamyl alcohol, which was not present in the control group. The content of 1-hexanol was significantly higher in PCT4 (559 μg/L) than in the other groups, and the other yeasts produced similar results (100–200 μg/L). In addition, PCT4 exhibited many unique volatile compounds, such as 1-heptanol, heptyl acetate and dimethyl phthalate, which were not produced by the other yeasts (Table 3). In addition, two terpenes, namely, Nerolidol and Citronellol, were detected in eight wine samples. The former was found in JCT3 (100.43 μg/L) and PCT4 (98.54 μg/L), while the latter was only detected in PCT4 (144.30 μg/L). The reason for the different terpene compounds in wine samples treated with different yeasts might be the different hydrolysis ability of glycosidases on different substrates [15].

**Figure 4:**
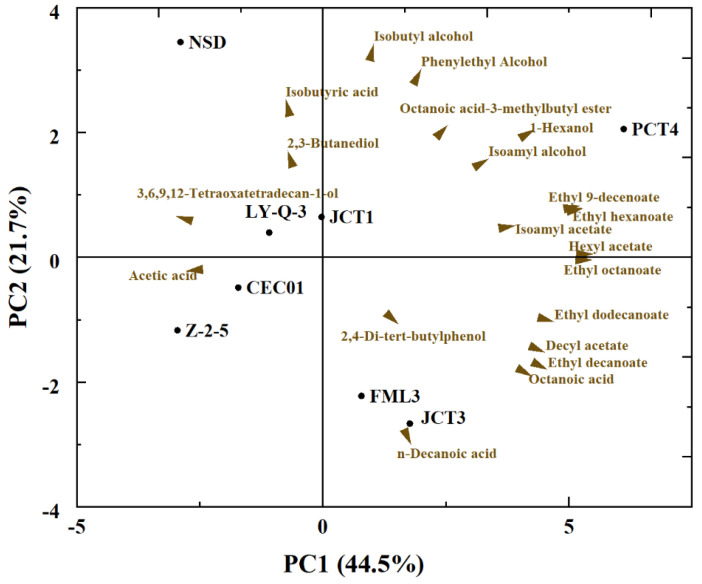
Principal component analysis (PCA) visualizing the relationships among yeasts and volatile compounds for wines.

PCA was performed using the main chemical data of wines obtained with each yeast to better compare and visualise the relationship between different yeasts and volatile compounds produced in Cabernet Sauvignon (Figure 4). Only volatile compounds with significant differences among most wines were used for analysis (*p* < 0.05). The first two principal components (PCs) accounted for 66.2% of the variation in the volatile compounds, with PC1 explained 44.5% of the contribution rate and PC2 explained 21.7%. The PC1 separated the wine samples based on different volatile compositions and esters (ethyl 9-decenoate, ethyl dodecanoate, ethyl hexanoate and ethyl octanoate) and was placed on the right side of PC1. The top of PC2 separated the wine samples based on alcohols (isobutyl alcohol, 1-hexanol and phenylethyl alcohol), and the bottom of PC2 separated the samples based on some acids (n-decanoic acid and octanoic acid). PCA displayed a clear different among isolates, especially the isolates of PCT4, JCT3 and FML3. FML3 and JCT3, identified as *S. cerevisiae* at the species level, had a positive correlation with n-decanoic acid. PCT4 had the most kinds of volatile compounds and differed from the other isolates because it was mainly positively correlated with ethyl 9-decenoate, ethyl hexanoate, 1-hexanol, hexyl acetate and ethyl octanoate. Similar to the results in Table 2, Z-2-5 had a greater content of acids, which explained why it was significantly different from the other yeasts. Therefore, these isolated yeasts showed biodiversity at the strain level.

## 4. Conclusions

In this study, native yeasts were isolated from four representative wine-producing regions in China and identified by molecular biology. The environmental tolerance and fermentation characteristics of the isolated yeasts were evaluated. *S. cerevisiae,* Hanseniaspora, and *Metschnikowia* were the most representative species, accounting for 90% of the isolates. Many yeasts showed fermentation potential through different screening trials, which highlighted inter- and intraspecies differences. *S. cerevisiae* had good tolerance, especially ethanol tolerance, but it produced H_2_S, which has a negative effect on wine. *Hanseniaspora* demonstrated good enzyme activity, which could enhance the complexity of wine aroma. In particular, the sequential fermentation of PCT4 and commercial *S. cerevisiae* produced richer aromas than the other isolates and synthesised special volatile compounds (such as Ethyl heptanoate, 1-Heptanol, Heptyl acetate and Dimethyl phthalate) that were absent in commercial yeasts. Hence, these yeasts could be used for fermentation with typicality and individuality for wine, helping to alleviate the phenomenon of wine homogenization. This work has significant implications for the wine making industry. Further fermentation testing and sensory analysis would be a fruitful direction for future research.

## Figures and Tables

**Table 1:** Different strains distribution of code, yeast species, source and region.

Serial No.	Code of the Strains	Yeast Species	Source	Region
1	FMS3	*Saccharomyces cerevisiae*	Grape Epidermis	Huailai
2	CC3	*Hanseniaspora uvarum*	Grape Epidermis	Huailai
3	CXD3	*Rhodotorula mucilaginosa*	Grape Epidermis	Huailai
4	FML3	*Saccharomyces mikatae*	Grape Epidermis	Huailai
5	SZ2	*Hanseniaspora uvarum*	Grape Epidermis	Huailai
6	SXL4	*Rhodotorula mucilaginosa*	Grape Epidermis	Huailai
7	PXD4	*Hanseniaspora pseudoguilliermondii*	Grape Epidermis	Penglai
8	CXS3	*Hanseniaspora uvarum*	Grape Epidermis	Huailai
9	PXD1	*Rhodotorula mucilaginosa*	Grape Epidermis	Penglai
10	PMS3	*Hanseniaspora pseudoguilliermondii*	Grape Epidermis	Penglai
11	JCT3	*Saccharomyces cerevisiae*	Vineyard Soil	Yili
12	JCT1	*Hanseniaspora pseudoguilliermondii*	Vineyard Soil	Yili
13	PCT4	*Hanseniaspora pseudoguilliermondii*	Vineyard Soil	Penglai
14	CXST5	*Hanseniaspora pseudoguilliermondii*	Vineyard Soil	Huailai
15	Q-1-3	*Hanseniaspora uvarum*	Spontaneous Fermentation	Qingtongxia
16	Q-1-1	*Hanseniaspora uvarum*	Spontaneous Fermentation	Qingtongxia
17	Q-2-2	*Hanseniaspora uvarum*	Spontaneous Fermentation	Qingtongxia
18	Z-1-1	*Hanseniaspora uvarum*	Spontaneous Fermentation	Qingtongxia
19	Z-2-1	*Saccharomyces cerevisiae*	Spontaneous Fermentation	Qingtongxia
20	Z-1-2	*Metschnikowia sinensis*	Spontaneous Fermentation	Qingtongxia
21	Z-2-5	*Hanseniaspora uvarum*	Spontaneous Fermentation	Qingtongxia
22	H-1-1	*Saccharomyces cerevisiae*	Spontaneous Fermentation	Qingtongxia
23	H-2-1	*Saccharomyces cerevisiae*	Spontaneous Fermentation	Qingtongxia
24	LY Z-4	*Hanseniaspora uvarum*	Spontaneous Fermentation	Huailai
25	LY Z-5	*Hanseniaspora uvarum*	Spontaneous Fermentation	Huailai
26	CX-Q-3	*Metschnikowia sinensis*	Spontaneous Fermentation	Huailai
27	CX-Q-4	*Metschnikowia sinensis*	Spontaneous Fermentation	Huailai
28	LY Q-3	*Hanseniaspora uvarum*	Spontaneous Fermentation	Huailai
29	LY Q-2	*Hanseniaspora pseudoguilliermondii*	Spontaneous Fermentation	Huailai
30	CX-Z-3	*Metschnikowia sinensis*	Spontaneous Fermentation	Huailai
31	CX-Z-2	*Metschnikowia sinensis*	Spontaneous Fermentation	Huailai

**Table 2:** Oenological analysis of the wines fermented by commercial and isolated yeasts. Data are means ± standard deviations. a–h: Different letters in the same column represent significant differences (*p* < 0.05) according to the Duncan test. For non-*Saccharomyces* yeasts (NSD, Z-2-5, PCT4, JCT1 and LY Q-3), sequential fermentation was completed by combining them with commercial *S. cerevisiae* CEC01.

Strain	Ethanol (%/*v*/*v*)	Reducing Sugar (g/L)	Total Acidity (g/L)	pH
CEC01	12.23 ± 0.11 d	2.87 ± 0.12 cde	7.25 ± 0.22 bc	3.47 ± 0.01 d
NSD	14.15 ± 0.03 b	2.67 ± 0.15 e	7.13 ± 0.01 bc	3.60 ± 0.01 b
Z-2-5	9.49 ± 0.12 h	3.33 ± 0.23 a	8.47 ± 0.33 a	3.22 ± 0.01 g
PCT4	10.41 ± 0.11 g	3.17 ± 0.06 ab	6.88 ± 0.22 cd	3.50 ± 0.01 c
JCT1	13.65 ± 0.03 c	2.73 ± 0.12 de	7.38 ± 0.22 b	3.61 ± 0.01 b
LY Q-3	14.45 ± 0.08 a	2.60 ± 0.08 e	7.00 ± 0.22 bcd	3.69 ± 0.02 a
JCT3	10.72 ± 0.03 f	3.13 ± 0.25 abc	6.38 ± 0.38 e	3.40 ± 0.01 f
FML3	11.80 ± 0.02 e	2.97 ± 0.15 bcd	6.63 ± 0.22 de	3.43 ± 0.01 e

**Table 3:** Identification and relative contents (μg/L) of volatile compounds of the different yeasts in Cabernet Sauvignon wine. a–h: Different letters in the same row represent significant differences (*p* < 0.05) according to the Duncan test. For non-*Saccharomyces* yeasts (NSD, Z-2-5, PCT4, JCT1 and LY Q-3), their sequential fermentation with CEC01 is presented; For *S. cerevisiae* (CEC01, FML3 and JCT3), their monoculture fermentation is presented.

Volatile Compounds	Yeast Isolate							
	CEC01	FML3	JCT3	JCT1	LY Q-3	NSD	PCT4	Z-2-5
Alcohols (17)								
Isobutyl alcohol	297.17 ± 26.58 c	109.57 ± 17.26 e	130.40 ± 31.19 d	565.28 ± 36.82 b	568.51 ± 22.28 b	568.14 ± 24.63 b	682.38 ± 34.63 a	286.80 ± 13.89 c
Phenylethyl alcohol	15,020.65 ± 97.62 d	14,685.22 ± 58.23 e	13,757.56 ± 123.36 f	15,623.59 ± 105.79 c	13,136.80 ± 71.83 g	25,171.26 ± 125.97 a	23,306.94 ± 142.35 b	7904.71 ± 77.15 h
Isoamyl alcohol	-	4843.99 ± 79.02 d	3908.22 ± 53.33 e	6776.87 ± 46.66 b	-	5887.31 ± 78.11 c	7694.14 ± 76.71 a	-
2-Dodecanol	-	-	35.24 ± 2.12 a	-	-	-	-	-
1-Decanol	-	-	62.02 ± 5.81 b	-	-	-	98.47 ± 3.04 a	-
1-Heptanol	-	-	-	-	-	-	102.69 ± 6.49 a	-
1-Hexanol	199.24 ± 6.86 c	118.24 ± 9.07 f	132.18 ± 3.02 e	285.65 ± 1.85 b	177.87 ± 3.51 d	171.12 ± 2.34 d	559.00 ± 5.31 a	107.35 ± 3.85 g
2,3-Butanediol	-	-	46.10 ± 1.88 e	-	635.46 ± 21.15 a	298.05 ± 35.59 b	176.44 ± 7.94 c	68.22 ± 1.22 d
Benzyl alcohol	16.92 ± 0.77 c	-	-	135.17 ± 3.59 a	-	-	38.64 ± 1.30 b	-
Nerolidol	-	-	100.43 ± 1.85 a	-	-	-	98.54 ± 2.68 a	-
3-Heptanol-6-methyl	-	-	-	-	-	486.14 ± 15.92 b	1668.85 ± 60.26 a	-
3-Heptanol-5-methyl	-	-	-	-	-	329.96 ± 42.09 a	-	-
1-Propanol-3-(methylthio)	-	-	-	-	-	257.95 ± 43.94 a	101.38 ± 4.31 b	-
Octaethylene glycol	6.11 ± 0.17 a	-	1.02 ± 0.10 b	-	-	-	-	-
3,6,9,12-Tetraoxatetradecan-1-ol	6.81 ± 0.23 e	28.94 ± 0.31 c	-	35.06 ± 2.25 b	-	12.99 ± 0.79 d	-	103.77 ± 4.71 a
Citronellol	30.60 ± 7.75 b	-	-	-	-	-	144.30 ± 10.21 a	-
3,6,9,12,15-Pentaoxanonadecan-1-ol	-	23.52 ± 1.42 b	-	-	-	-	40.40 ± 2.21 a	-
Esters (23)								
7-Octenoic acid-ethyl ester	-	-	-	-	-	-	157.25 ± 14.41a	-
Isoamyl acetate	603.97 ± 69.94 f	747.38 ± 45.74 e	574.09 ± 30.46 g	1071.95 ± 70.88 c	839.24 ± 51.41 d	139.04 ± 9.83 h	2319.97 ± 108.92 a	1301.11 ± 56.09 b
Ethyl hexanoate	803.67 ± 58.48 d	1091.76 ± 76.77 b	952.94 ± 45.18 c	813.32 ± 63.26 d	552.95 ± 43.21 e	541.55 ± 35.25 e	2670.64 ± 128.42 a	401.73 ± 23.83 f
Ethyl heptanoate	-	-	-	-	-	-	51.71 ± 3.29 a	-
Ethyl octanoate	4584.64 ± 78.65 f	7343.63 ± 178.26 c	8814.86 ± 201.71 b	6641.95 ± 210.55 e	6897.41 ± 185.11 d	1649.47 ± 73.15 h	15,503.41 ± 229.64 a	2752.36 ± 105.64 g
Octanoic acid-3-methylbutyl ester	-	127.35 ± 9.95 c	214.50 ± 22.86 b	-	-	-	265.17 ± 15.31 a	71.34 ± 2.79 d
Isopentyl formate	6258.74 ± 217.13 a	-	-	-	5027.63 ± 188.71 b	-	-	3382.72 ± 155.58 c
Heptyl acetate	-	-	-	-	-	-	44.50 ± 2.35 a	-
Hexyl acetate	69.05 ± 7.07 e	127.64 ± 21.13 c	161.49 ± 9.09 b	134.92 ± 7.93 c	97.40 ± 12.07 d	-	333.45 ± 24.54 a	37.30 ± 3.73 f
Diethyl succinate	29.58 ± 1.62 b	-	-	-	-	-	68.72 ± 1.46 a	-
Ethyl dodecanoate	139.46 ± 9.03 g	1169.91 ± 21.08 b	1127.46 ± 32.10 c	365.33 ± 8.93 e	990.83 ± 82.29 d	-	1455.42 ± 99.04 a	160.14 ± 11.66 f
n-Capric acid isoamyl ester	-	-	-	-	-	-	167.54 ± 24.01 a	-
Ethyl acetate	-	-	-	-	1552.18 ± 91.17 b	-	-	7997.75 ± 215.76 a
Dimethyl phthalate	-	-	-	-	-	-	171.99 ± 39.18 a	-
Isobutyl acetate	-	-	-	-	-	-	-	71.73 ± 12.92 a
Isopentyl hexanoate	-	-	19.62 ± 1.67 b	-	-	-	82.78 ± 4.82 a	-
Ethyl decanoate	3111.23 ± 90.45 f	8299.81 ± 218.35 c	10,332.24 ± 236.91 a	4689.98 ± 104.89 d	3524.60 ± 96.06 e	1220.77 ± 87.12 h	8400.57 ± 244.24 b	1601.23 ± 94.26 g
Ethyl 9-decenoate	2491.42 ± 105.99 f	4349.97 ± 177.99 d	5140.99 ± 210.13 c	5714.03 ± 192.47 b	3935.68 ± 124.99 e	622.51 ± 106.64 h	16,502.11 ± 251.01 a	1679.09 ± 96.47 g
Ethyl phenylacetate	25.12 ± 3.86 b	-	31.14 ± 1.61 a	20.52 ± 1.94 c	-	-	-	-
n-Caprylic acid isobutyl ester	-	-	-	-	-	-	88.02 ± 1.72 a	-
Decyl acetate	1161.61 ± 78.82 f	2533.63 ± 76.71 c	2750.71 ± 95.12 a	1839.29 ± 54.82 e	2195.52 ± 57.77 d	760.08 ± 40.55 h	2639.27 ± 62.51 b	1002.96 ± 46.23 g
Butanoic acid-4-hydroxy	-	-	-	-	-	-	149.08 ± 15.17 a	-
Acids (6)								
Octanoic acid	859.09 ± 51.81 e	1542.39 ± 56.45 c	2255.74 ± 93.73 a	742.06 ± 46.32 f	-	-	2103.63 ± 107.18 b	996.78 ± 28.93 d
9-Decenoic acid	-	-	481.25 ± 22.56 b	-	-	-	607.81 ± 58.57 a	-
Hexanoic acid	-	-	273.08 ± 22.18 b	-	-	-	509.04 ± 35.09 a	220.01 ± 15.05 c
Isobutyric acid	-	-	79.93 ± 27.27 b	38.99 ± 0.73 c	-	272.89 ± 14.88 a	73.47 ± 5.81 b	20.03 ± 2.35 d
n-Decanoic acid	430.49 ± 17.01 f	1686.18 ± 59.12 b	2988.63 ± 91.87 a	468.91 ± 106.48 e	-	-	630.23 ± 30.35 d	999.89 ± 67.36 c
Acetic acid	176.88 ± 5.46 g	79.49 ± 3.81 h	205.93 ± 4.21f	249.36 ± 1.04 e	1402.80 ± 60.44 b	1067.86 ± 64.19 c	342.12 ± 16.39 d	4798.10 ± 93.27 a
Ethers (4)								
15-Crown-5	-	26.97 ± 0.66 a	-	-	-	-	-	-
18-crown-6	-	15.62 ± 2.33 b	7.36 ± 0.02 c	62.77 ± 2.89 a	-	-	-	-
Octaethylene glycol monododecyl ether	-	35.78 ± 1.94 a	-	-	-	-	-	-
Aldehydes (2)								
Acetaldehyde	-	-	-	-	-	-	-	41.15 ± 2.71 a
Benzaldehyde	-	-	-	175.21 ± 11.46 a	-	-	176.93 ± 5.59 a	-
Volatile phenols (2)								
Phenol-2,6-bis(1,1-dimethylethyl)	-	107.96 ± 8.08 a	-	-	-	-	-	-
2,4-Di-tert-butylphenol	-	131.40 ± 9.71 d	240.50 ± 1.38 c	-	-	-	354.90 ± 7.73 b	523.51 ± 4.54 a
Ketones (1)								
2-Undecanone	-	-	35.19 ± 2.43 a	-	-	-	-	-

## Data Availability

The data used to support the findings of this study can be made available by the corresponding author upon request.

## Supplementary Materials

The following supporting information can be downloaded at: https://www.mdpi.com/article/10.3390/foods12163073/s1, Table S1: Growth performance of selected yeasts in different stress conditions.
