# Supplementary material for: Evaluation of Indigenous Yeasts Screened from Chinese Vineyards as Potential Starters for Improving Wine Aroma
Source: Foods. 2023 Aug 16;12(16):3073. doi: 10.3390/foods12163073 (PMC10453611; doi:10.3390/foods12163073)
Supplement: Supplementary file 1 [file foods-12-03073-s001.zip › foods-2502274-supplementary.pdf]

Supplementary Material

Supplementary Table: Growth performance of selected yeasts in different stress conditions

Table S1 Growth performance of selected yeasts in different stress conditions.

Stress conditions	FMS3	JCT3	FML3	Z-2-1	H-1-1	H-2-1
control	++++	++++	++++	++++	++++	++++
V2	++++	++++	++++	++++	++++	++++
V4	++++	++++	++++	++++	++++	++++
V6	++++	++++	++++	++++	++++	++++
V8	++++	++++	++++	++++	++++	++++
V10	++++	++++	++++	++++	++++	++++
V12	++++	++++	++++	++++	++++	++++
V14	++++	+++	+	+	-	-
V16	++	-	-	-	-	-
G20	++++	++++	++++	++++	++++	++++
G30	++++	++++	++++	++++	++++	++++
G40	++++	++++	++++	++++	++++	++++
G50	++++	++++	++++	++++	++++	++++
pH3.1	++++	++++	++++	++++	++++	++++
pH3.3	++++	++++	++++	++++	++++	++++
pH4.3	++++	++++	++++	++++	++++	++++
S50	++++	++++	++++	++++	++++	++++
S100	++++	++++	++++	++++	++++	++++
S150	++++	++++	++++	++++	++++	++++
S200	++++	++++	++++	++++	++++	++++
S250	++++	++++	++++	++++	++++	++++

(continued on next page)

Table S1 (countined)

Stress conditions	JCT1	SZ2	SXL4	PXD4	PCT4	PXD1	PMS3	CXS3	CC3	CXD3	CXST5	Q-1-3	Q-1-1	Q-2-2
control	++++	++++	++++	++++	++++	++++	++++	++++	++++	++++	++++	++++	++++	++++
V2	++++	++++	-	++++	++++	+	++++	++++	++++	++	++++	++++	++++	++++
V4	++++	++++	-	++++	++++	-	++++	++++	++++	-	++++	++++	++++	++++
V6	++++	-	-	-	-	-	-	++++	-	-	-	-	++++	-
V8	+++	-	-	-	-	-	-	-	-	-	-	-	-	-
V10	-	-	-	-	-	-	-	-	-	-	-	-	-	-
G20	++++	++++	+	++++	++++	++++	++++	++++	++++	++	++++	++++	++++	++++
G30	++++	++++	-	++++	++++	++	++++	++++	++++	+	++++	++++	++++	++++
G40	++++	++++	-	++++	++++	+	++++	++++	++++	-	++++	++++	++++	++++
G50	++++	++++	-	++++	++++	-	++++	++++	++++	-	++++	++++	++++	++++
pH3.1	++++	++++	+++	++++	++++	++++	++++	++++	++++	+++	++++	++++	++++	++++
pH3.3	++++	++++	+++	++++	++++	++++	++++	++++	++++	+++	++++	++++	++++	++++
pH4.3	++++	++++	+++	++++	++++	++++	++++	++++	++++	+++	++++	++++	++++	++++
S50	++++	++++	-	++++	++++	++++	++++	++++	++++	-	++++	++++	++++	++++
S100	++++	++++	-	++++	++++	-	++++	++++	++++	-	++++	++++	++++	++++
S150	++++	++	-	++++	+++	-	++++	++++	+++	-	++++	++++	+++	++++
S200	++++	++	-	+++	+++	-	+++	++++	+	-	+++	++++	+	++++
S250	++++	-	-	-	+++	-	++	++++	-	-	+++	++++	++++	+

(continued on next page)

Table S1 (countined)

Stress conditions	Z-1-1	Z-1-2	Z-2-5	LY-Z-4	LY-Z-5	LY-Q-3	LY-Q-2	CX-Z-3	CX-Z-2	CX-Q-3	CX-Q-4
control	++++	++++	++++	++++	++++	++++	++++	++++	++++	++++	++++
V2	++++	++++	++++	++++	++++	++++	++++	++++	++++	++++	++++
V4	++++	++++	++++	++++	++++	++++	++++	+++	++++	+	++++
V6	-	++	-	-	-	-	-	-	-	-	+
V8	-	-	-	-	-	-	-	-	-	-	-
V10	-	-	-	-	-	-	-	-	-	-	-
G20	++++	++++	++++	++++	++++	++++	++++	++++	++++	++++	++++
G30	++++	++++	++++	++++	++++	++++	++++	++++	++++	++++	++++
G40	++++	++++	++++	++++	++++	++++	++++	++++	++++	++++	++++
G50	++++	++++	++++	++++	++++	++++	++++	++++	++++	++++	++++
pH3.1	++++	++++	++++	++++	++++	++++	++++	++++	++++	++++	++++
pH3.3	++++	++++	++++	++++	++++	++++	++++	++++	++++	++++	++++
pH4.3	++++	++++	++++	++++	++++	++++	++++	++++	++++	++++	++++
S50	++++	++++	++++	++++	++++	++++	++++	++++	++++	++++	++++
S100	++++	++++	++++	++++	++++	++++	++++	++++	++++	++++	++++
S150	+++	++++	++++	+++	+++	++	++++	++++	++++	++++	++++
S200	+++	++++	+	+++	+++	-	++++	++++	++++	++++	++++
S250	+++	++++	+	++	+++	-	++	++++	++++	++++	++++

++++: full gas in the durham; +++: 2/3 gas; ++: 1/3 gas; +: 1/5 gas; -: no gas

V2-V10: 2%-10% concentrations of ethanol; G20-G50: 20%-50% concentrations of glucose; S50-S250: 50-250mg/L concentrations of free SO_2
